# Recognizing the authenticity of emotional expressions: F0 contour matters when you need to know

**DOI:** 10.3389/fnhum.2014.00144

**Published:** 2014-03-11

**Authors:** Matthis Drolet, Ricarda I. Schubotz, Julia Fischer

**Affiliations:** ^1^Cognitive Ethology Laboratory, German Primate CenterGöttingen, Germany; ^2^Biological Psychology, University of MünsterMünster, Germany; ^3^Minerva Group Motor Cognition, Max Planck Institute for Neurological ResearchKöln, Germany

**Keywords:** emotion, speech, prosody, theory of mind, context, authenticity

## Abstract

Authenticity of vocal emotion expression affects emotion recognition and brain activity in the so-called Theory of Mind (ToM) network, which is implied in the ability to explain and predict behavior by attributing mental states to other individuals. Exploiting the variability of the fundamental frequency (F0 contour), which varies more (higher contour) in play-acted expressions than authentic ones, we examined whether contour biases explicit categorization toward a particular authenticity or emotion category. Moreover, we tested whether contour modulates blood-oxygen-level dependent (BOLD) response in the ToM network and explored the role of task as a top-down modulator. The effects of contour on BOLD signal were analyzed by contrasting high and low contour stimuli within two previous fMRI studies that implemented emotion and authenticity rating tasks. Participants preferentially categorized higher contour stimuli as play-acted and lower contour stimuli as sad. Higher contour was found to up-regulate activation task-independently in the primary auditory cortex. Stimulus contour and task were found to interact in a network including medial prefrontal cortex, with an increase in BOLD signal for low-contour stimuli during explicit perception of authenticity and an increase for high-contour stimuli during explicit perception of emotion. Contour-induced BOLD effects appear to be purely stimulus-driven in early auditory and intonation perception, while being strongly task-dependent in regions involved in higher cognition.

## INTRODUCTION

Emotions play a fundamental role in human social behavior. Within an evolutionary framework, emotions are considered to be evolved, adaptive mechanisms that facilitate an organism’s coping with important events (Darwin, 1872; Scherer, 2000). Although the dispute about the nature of emotions is far from settled, there is a growing consensus that emotion should be viewed as a multi-component entity (Scherer, 1984; Frijda, 1986; Lazarus, 1991). The three major components of emotion are neurophysiological response patterns, subjective feelings, and the motor patterns of expressions of emotions.

Emotions can be expressed through body language (Van den Stock et al., 2007), the face (DeKosky et al., 1980; Ekman et al., 1990), or the voice (Scherer, 1991; Wurm et al., 2001). A key question in understanding emotions and their expression is whether emotions constitute graded or discrete entities (Hamann, 2012; Truong et al., 2012). The discrete model initially gained support from research into facial emotion expressions (Söderling, 1959; Freedman, 1964; Ekman, 1992). Researchers that primarily investigated emotion expression in the voice, however, lean toward a graded model in which emotions and their expressions vary along continuous dimensions, including valence and arousal; some models also include potency (Scherer, 1984). This concurs with the fact that vocal expressions of emotion vary along continuous dimensions, such as pitch and intensity (Banse and Scherer, 1996). These multidimensional models have also been applied to the perception of vocal expressions of emotion (Truong et al., 2012; Witteman et al., 2012), implying that the patterns revealed in emotion perception map onto the expression of emotions. Thus, studying the perception of emotion expressions can help us understand the nature of emotions themselves.

Neuroscientific studies have provided evidence for the different theoretical accounts of emotions. Panksepp (1998) argued that the discovery of brain circuits and regions such as the amygdala that are associated with specific emotional responses and perception of facial emotional expressions supports a discrete model of emotion. Adolphs et al. (2002) and Adolphs (1999), however, found that the amygdala was not necessary for the perception of vocal emotion expression. Instead, the superior and middle temporal regions are involved in the perception of intonation or prosody (Vigneau, 2006; Wildgruber et al., 2006; Wiethoff et al., 2008). This activation extends further into the temporal cortex than just the primary auditory cortex, also known as the transverse temporal gyrus (TTG), which is the region where cortical auditory processing begins (Celesia, 1976; Binder et al., 1994). While these regions can be differentially activated by various expressions of emotion (Ethofer et al., 2009), the evidence for clearly distinct regions for perceiving expressions of different emotions is still debated.

Another core issue is the link between emotion and cognition at the level of the experience, expressions, and perception of emotion. Buchanan et al. (2000) observed that activity in the inferior frontal cortex was enhanced during explicit recognition of emotional prosody as compared to just listening to emotional prosody (see also Adolphs et al., 2002). This led to a model in which the superior temporal and inferior frontal cortices are involved in basic acoustic and task-dependent processing, respectively (Schirmer and Kotz, 2006). Additionally, the orbitofrontal (OFC) and anterior cingulate cortices (ACC) may be central to making evaluative decisions based on the emotions associated with available choices (Bechara et al., 2000) during the perception of emotional prosody (Bach et al., 2008). Such evaluation is included in models of emotion as so-called *cognitive appraisal*, often including information from different modalities and/or previous experiences. As such, the perception of emotion can be modified by context through cognitive appraisal of the situation and environment in which that expression is produced or perceived (Schirmer et al., 2006; Barrett and Kensinger, 2010; Brück et al., 2011). These findings indicate that the perception of emotional expressions parallels the multi-component dimensional nature implied in their production (Scherer, 1984; Lazarus, 1991).

A particularly relevant contextual modulator of emotion perception is recognition of speaker intention (Drolet et al., 2012, 2013). The ability to perceive another’s intention is commonly called Theory of Mind (ToM; Premack and Woodruff, 1978), defined as the implicit or explicit attribution of mental states (e.g., desires, beliefs, and intentions) to others and self (Frith and Singer, 2008; Frith and Frith, 2012). ToM has been studied extensively, including its evolutionary roots (Povinelli and Preuss, 1995), its development (Wimmer and Perner, 1983; Rakoczy and Tomasello, 2006), and its every-day use in humans (Tomasello, 2003; Abraham et al., 2008). Importantly, ToM has been shown to both interact with emotion perception and be influenced by it (Mier et al., 2010; Ray and Zald, 2012). For example, Blair et al. (2007) showed that active regulation of emotional distractors is required in cognitive tasks such as ToM. The influence of ToM on emotion recognition, however, has not been studied extensively.

Our previous work (Drolet et al., 2012) showed that emotional authenticity affects emotion perception. Recordings of speech produced by professional actors after instruction to express a specific emotion (hereafter play-acted) were compared to speech produced without external instruction (hereafter authentic). While authenticity affected the recognition of categories of emotion, there were no significant effects of arousal or valence dimensions, likely due to the subtle differences inherent to short vocal expressions. Explicit rating of authenticity did induce blood-oxygen-level dependent (BOLD) response modulation in the ToM network (medial prefrontal, retrosplenial and temporoparietal cortices) more so than did emotion categorization, while authentic stimuli additionally up-regulated activation in an important component of the ToM network, the medial prefrontal cortex (mPFC). A subsequent study required only an emotion categorization task (Drolet et al., 2013), while subjects were told via cues whether a recording was authentic or play-acted. In that study, instead of authentic stimuli up-regulating mPFC, play-acted stimuli up-regulated temporoparietal junction (TPJ), early auditory processing in TTG, and early sentence perception in middle temporal gyrus (MTG) and superior temporal gyrus (STG). Cueing influenced brain activation in ACC when there was a conflict between cue and authenticity of stimulus, but did not affect brain activation found in the previous study or emotion recognition. Taken together, our previous results indicate an interaction between bottom-up and top-down influences. While activation in early auditory cortices appears to be stimulus-dependent, BOLD signal in frontal regions appeared to increase for authentic stimuli during recognition of authenticity and for play-acted stimuli during recognition of emotion.

**FIGURE 1:**
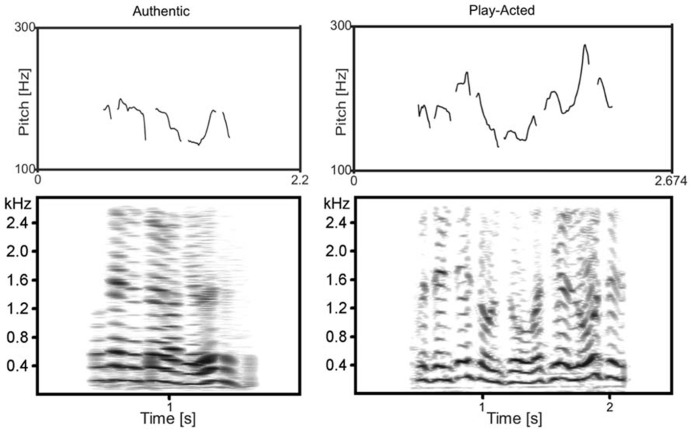
**F0 contour (top) and spectrogram (bottom) of two complementary example recordings.** Examples are of the same text (“… Präsenz ist und so, was hier los ist…” translated: “…presence is and such, what is going on here…”) from the original authentic and play-acted versions.

However, some important questions remain. Emotion is known to affect vocal expressions (Williams and Stevens, 1972; Banse and Scherer, 1996), but the acoustic properties that led to the aforementioned bottom-up effects of authenticity remain unclear. Jürgens et al. (2011) examined the acoustic correlates of authenticity and found that contour (i.e., variability of the fundamental frequency or F0 contour) was significantly greater for play-acted recordings than for authentic. The variability in fundamental frequency is measured across the entire span of the vocal expression (see **Figure 1**). So someone hearing an expression with *higher* variability may consider it more likely to be *play-acted*, while one with *lower* variability would be more likely to be *authentic*. The perception of such acoustical differences is paralleled by stimulus-induced activation in the brain. Wiethoff et al. (2008) showed that several acoustic parameters, including contour, correlated positively with activation in right STG, while a recent review by Vigneau et al. (2011) found that superior temporal and lateral frontal areas are activated by phonological processing.

Against this backdrop, we wished to determine whether F0 contour affects behavior and BOLD response when listening to emotional prosody and whether it is the acoustic variable that is responsible for the authenticity effects seen in the previous studies (Drolet et al., 2012, 2013). Three main questions were addressed: (1) whether F0 contour influences the recognition of authenticity, (2) whether it influences early auditory and intonation processing and the ToM network during explicit categorization of emotions, and (3) whether it up-regulates TPJ and mPFC activity dependent on whether participants are rating emotional category or authenticity. These questions were tested on both previously published datasets (Drolet et al., 2012, 2013). While the 2012 study included both the authenticity and emotion tasks, it became clear during the analysis that perception of emotional content required more power to be analyzed in detail. By combining the two studies it was possible to analyze the effects specific to the emotion task in the 2013 study (due to more emotion task trials), while contrasting task effects in the 2012 study. The studies included two different groups of participants and differences in the control tasks, but were otherwise setup identically. Based on the two previous studies, we predicted low contour stimuli would be rated as sad or authentic and high contour stimuli as anger or play-acted.

With regard to functional magnetic resonance imaging (fMRI), three hypotheses were tested. First, the effect of contour was analyzed parametrically to determine what, if any, region responded to the entire measurable span of contour. Based on differing contour between play-acted and authentic stimuli (Jürgens et al., 2011) and up-regulation in the primary auditory cortex by play-acted stimuli (Drolet et al., 2012), we expected stimuli with higher contour to induce higher activation in primary auditory cortex, STG and superior temporal sulcus.

Second, BOLD effects of extremely high and low contour values were identified in order to directly compare recordings that, based on contour, have a high probability of being either authentic or play-acted. Based on our previous studies, as well as Vigneau et al. (2011) and Wiethoff et al. (2008), we predicted that the BOLD response in STG, MTG, TPJ, mPFC and lateral frontal areas would increase with increasing contour.

Third, regions of interest (ROI) within the ToM network were examined in the previous contrast to determine whether task requirements modulate the effect contour has on the BOLD response. These ROIs were extracted from the two tasks in Drolet et al. (2012) to determine the interaction of bottom-up contour effects and top-down task effects. Based on previous results we expected authentic stimuli to up-regulate BOLD during authenticity tasks, and play-acted stimuli to do so during emotion tasks.

## MATERIALS AND METHODS

### STIMULUS SELECTION

Original recordings (mono wave format; sample rate of 44.1 kHz) were selected from German radio interviews of individuals talking in an emotional fashion (anger, fear, joy, sadness) about a highly charged ongoing or recollected event (e.g., the death of a child, winning a lottery, threatened by a current danger). Emotion was ascertained through verbal content and recording summaries. Staged and scripted settings were excluded. Of 80 speech segments, 35 were made outdoors but were of good quality with minimal background noise. To ensure inference-free verbal content, text-only transcripts were rated by naïve subjects. Recordings with emotion recognized better than chance were replaced to ensure neutral semantic content. The original set consisted of 80 recordings by 78 speakers (half male, half female; mean 1.75s ± 1.00 SD; range 0.36–4.06 s).

Play-acted stimuli were performed by actors from Germany (42 actors each replicated a maximum of three recordings of equivalent emotional content), who were told to express each text in their own way, using the transcripts, summaries, and emotion (stimulus segments were not indicated and actors never heard the originals). Recording environment was varied while minimizing background noise, with 30 of 80 made outdoors (mean 1.76s ± 1.02 SD; range 0.38–4.84 s). Average amplitudes of all stimuli were equalized with Avisoft SASLab Pro Recorder 4.40 (Berlin, Germany). The final stimulus set consisted of 20 samples of joy and sadness, 22 samples of anger and 18 samples of fear, both for authentic and play-acted sets.

### PARTICIPANTS

#### Study 1

24 female participants (mean 24 years old; range 20–30 years; right-handed; German mother-tongue), without a history of neurological or psychological complications (including the use of psychiatric medication), were selected and contacted using the Cologne Max-Planck Institute (MPI) database for fMRI experiments. Participants were informed about the potential risks of magnetic resonance imaging and screened by a physician. They gave informed consent before participating and were paid afterward. The experimental standards were approved by the local ethics committee and data were handled pseudonymously.

#### Study 2

Selection criteria were identical to Experiment 1, with 18 female participants selected (20–30 years, mean 24 years, right-handed, German mother-tongue).

### TRIAL AND STIMULUS PRESENTATION

#### Study 1

The program NBS Presentation (Neurobehavioral Systems, Inc., Albany, CA, USA) controlled the trial structure, timing, and order of each experimental run. Each run (one per participant) included 178 trials, of which 72 were used for an emotion rating task and 72 for an authenticity rating task. In addition, two control tasks were included: 16 word detection trials, in which participants had to count occurrences of the word “und” (“and”), and 18 empty trials with pink-noise playback. For emotion ratings four responses were possible: anger, sadness, happiness, fear (presented in German as: “Wut,” “Trauer,” “Freude,” “Angst”), while for authenticity ratings responses were authentic (“echt”) and play-acted (“theater”; described to participants beforehand as “gespielt,” i.e., play-acted). To minimize eye movement the maximal line-of-sight angle for visual information was kept under 5°. Trial type and stimulus type pseudo-randomizations were performed using conan (UNIX shell script: MPI for Neurology in Leipzig, Germany) to reduce any systematic effects that could have otherwise occurred with simple randomization. Each participant was shown a button sequence on-screen (800 × 600 pixel video goggles: NordicNeuroLab, Bergen, Norway) complementary to the response box layout (10 cm × 15 cm × 5 cm gray plastic box with a row of four black plastic buttons). For both emotion rating and word detection all buttons were assigned a possible response. For authenticity rating only the two left-most buttons were used.

#### Study 2

Software, hardware, and trial and stimulus randomization were identical to Experiment 1, but only the emotion rating task was applied (144 trials). One third (*n* = 48) were not cued (no authenticity information was provided), one third were cued as authentic, and one third were cued as play-acted. Cueing was congruent half the time and was presented above the response options as authentic or play-acted (“echt” or “spiel” respectively). The remaining 30 trials were used to implement two independent control tasks: 18 empty trials with pink-noise playback and 16 age task trials in which participants had to determine the age of the speaker (20, 30, 40, or 50).

### EXPERIMENTAL PROCEDURE

#### Study 1

Participants were fitted with headphones for audio playbacks (NNL: NordicNeuro-Lab, Bergen, Norway) after they were placed in a supine position on the fMRI table. Imaging was performed with a 3T Siemens MAGNETOM TrioTim (Cologne, Germany) system equipped with a standard birdcage head coil. Participants were placed with their four fingers (excluding thumb) positioned on the response buttons of the response box. Form-fitting cushions were utilized to prevent head, arm, and hand movements. Twenty-two axial slices (210 mm field of view; 64 × 64 pixel matrix; 4 mm thickness; 1 mm spacing; in-plane resolution of 3 mm× 3 mm) parallel to the bicommissural line (AC–PC) and covering the whole brain were acquired using a single-shot gradient echo planar imaging (EPI) sequence (2000 ms repetition time; 30 ms echo time; 90° flip angle; 1.8 kHz acquisition bandwidth) sensitive to BOLD contrast. In addition to functional imaging, 22 anatomical T1-weighted modified driven equilibrium fourier transform (MDEFT) images (Ugurbil et al., 1993; Norris, 2000) were acquired. In a separate session, high-resolution whole-brain images were acquired from each participant to improve the localization of activation foci using a T1-weighted 3D-segmented MDEFT sequence covering the whole brain. Functional data were mapped onto this 3D average using the 2D anatomical images made immediately following the experiment. Including a visual and auditory test prior to the experiment, one experimental run lasted approximately 45 min.

#### Study 2

The general procedure was identical to experiment 1. Twenty-four axial slices were acquired using a single-shot gradient EPI sequence sensitive to BOLD contrast.

### BEHAVIORAL STATISTICS

In order to determine whether contour has an effect on participant responses, the responses to both emotion and authenticity tasks from study 1 were examined as to whether labeled recordings differed significantly in contour values (correct and incorrect trials were analyzed together as this had no effect in previous analyses). The generalized linear model was implemented (R Statistical Package v2.15; R Development Core Team, 2008) to determine the best model fit for response rates with the glmer function from the lme4 package using restricted maximum likelihood estimation with binomial error structure and logit link function (Baayen et al., 2008). The basic model examined the effect contour has on responses with participant included as a random factor [glmer (Response ~ Contour + (1|Subject), family = binomial, REML = FALSE)]. *Post hoc* statistics were applied using a likelihood ratio test (LRT) with a Chi-squared distribution (χ^2^) to examine the effect on each emotion and were corrected for multiple comparisons.

### FUNCTIONAL MRI STATISTICS

After motion correction using Siemens rigid-body registration protocol (München, Germany), the functional data were processed using the software package LIPSIA v1.5.0 (Lohmann et al., 2001). This software package is available under the GNU General Public License (). To correct for temporal offset between the slices acquired in one image a cubic-spline interpolation was employed. Low-frequency signal changes and baseline drifts were removed using a temporal high-pass filter set for each scanned participant dependent on the pseudo-randomized design (filter frequency range: 1/75–85 Hz). Spatial smoothing was performed with a Gaussian filter of 5.65 mm full width at half maximum (FWHM) (sigma = 2.4). To align the functional data slices with a 3D stereotactic coordinate reference system, a rigid linear registration with 6° of freedom (three rotational, three translational) was applied. The rotational and translational parameters were acquired on the basis of the MDEFT slices to achieve an optimal match between these slices and the individual 3D reference dataset. The MDEFT volume dataset with 160 slices and 1-mm slice thickness was standardized to the Talairach stereotactic space (Talairach and Tournoux, 1988). The rotational and translational parameters were subsequently transformed by linear scaling to a standard size. The resulting parameters were then used to transform the functional slices using trilinear interpolation, so that the resulting functional slices were aligned with the stereotactic coordinate system, thus generating output data with a spatial resolution of 3 mm× 3 mm× 3 mm (27 mm × 27 mm × 27 mm).

Two design matrices were applied. In the first, contour (variation in fundamental frequency; measured as standard deviation) was modeled parametrically to examine its correlation with brain activation. The design matrix contained all authentic and play-acted stimulus trials in each of the first two event types respectively, with an amplitude vector of one. The second and third event types each also contained all authentic and play-acted stimulus trials with an amplitude vector corresponding to the specific stimulus’s contour value. The last event type in the design matrix, null-events, was assigned an amplitude value of one. This analysis was performed for the data from Drolet et al. (2012; with authenticity and emotion task) and Drolet et al. (2013; only with emotion task).

In order to further substantiate the parametric analysis, an additional step was taken. Using a second design, stimuli with extreme values of contour were contrasted directly. To do so, trials were preselected based on their respective recording’s contour value and grouped as high or low contour trials within the design matrix. Since we know authenticity correlates with contour (Jürgens et al., 2011), a simple block selection of upper and lower contour stimuli would lead to selections with unequal numbers of, and unbalanced mean contour values for, authentic and play-acted stimulus categories. Therefore, individual stimuli were excluded from each group to ensure equal numbers of authentic and play-acted stimuli (15 of each) and equivalent average contour values (**Table 1**). Exclusions were performed pseudo-randomly, such that stimuli that were not included affected the group-average contour values but did not affect any other parameter. Stimulus emotion was included as a regressor of no interest. High and low contour trial groups were contrasted for emotion task trials in both studies and authenticity task trials in study 1 (trials outside the low and high contour groups were included as a single regressor of no interest). Subsequently, a conjunction of the two contrasts from study 1 was used to examine regions activated in both tasks, while an exclusive disjunction was applied to uncover regions more highly activated in one task but not in the other.

**Table 1:** Mean values of high and low stimulus groups by contour (standard deviation of F0).

		Authentic (Hz)	Play-acted (Hz)	Statistics
Study 1	Low contour	7.03 ± 3.14	8.53 + 3.00	*t* = 2.07, *p* > 0.2
	High contour	48.44 ± 13.83	56.93 + 22.39	*t* = 2.04, *p* > 0.1
Study 2	Low contour	6.78 ± 3.00	8.27 + 2.99	*t* = 2.06, *p* > 0.2
	High contour	48.89 ± 15.24	56.36 + 20.75	*t* = 2.05, *p* > 0.1

* 
Mean contour (measured as standard deviation of fundamental frequency) and standard deviation of the selected high and low value recordings for each authenticity category and study. Statistics represent *t*-tests of the differences between the contour values by authenticity as applied to BOLD contrasts.*

Statistical evaluation was based on a least-squares estimation using the general linear model (GLM) for serially auto-correlated observations (Worsley and Friston, 1995; Friston et al., 1998). Both designs were generated with a delta function, convolved with the hemodynamic response function (gamma function). Each trial in the design matrix was identified by its onset time and stimulus length, while speaker repetition was included to prevent this from influencing the statistical analysis. Brain activations were analyzed time-locked to recording onset and the analyzed epoch was set individually for each trial to the duration of the respective stimulus. The model equation, including the observation data, design matrix, and error term, was convolved with a Gaussian kernel of dispersion 5.65 s FWHM to account for temporal autocorrelation (Worsley and Friston, 1995). In the following, contrast images (i.e., beta value estimates of the raw-score differences between specified conditions) were generated for each participant. As all individual functional datasets were aligned to the same stereotactic reference space, the single-subject contrast images were entered into a supplementary second-level analysis on the basis of Bayesian statistics (Neumann and Lohmann, 2003).

Bayesian statistics provide an alternative to frequentist significance tests. Instead of testing the estimated probability of detecting activation with the null hypothesis of no activation being true (i.e., *P*-values), the Bayesian approach directly infers the probability that a contrast between two conditions is greater than 0. When applied to second-level analyses, the probability that a contrast is larger than 0 is calculated based on the parameter estimations for the individual participants on the first level (i.e., the beta-values of the GLM). In the approach by Neumann and Lohmann (2003), posterior probability maps for the effects of interest are calculated on the basis of the resulting least-squares estimates of parameters for the GLM. The output of the Bayesian second-level analysis is a map integrating the reliability of activation differences between categories and the probability that the contrast is larger than 0 (percentage value between 0 and 100). Bayesian statistics consider only voxels for which signal was measured in all participants. A threshold of 99.5% and minimum cluster size of 100 voxels was applied to the probability maps and listed activation maxima. Bayesian inferences are not susceptible to problems of multiple comparisons thanks to direct computation of the probabilities.

Finally, the ROI analysis was performed on the high versus low contour contrast to determine the influence of contour relative to task instruction. ROIs were selected from the high versus low contour contrast from study 2 (Drolet et al., 2013), which was possible due to the higher power from the number of emotion tasks, to avoid statistical issues with so-called “double-dipping” (Kriegeskorte et al., 2009). GLM beta values (measure of BOLD response) within these ROIs were extracted separately for the two tasks in study 1 (Drolet et al., 2012). ROIs were selected from peak activations in mPFC bilaterally (*x,y,z* of Talairach space: 1,44,22; -2,47,24), and left TPJ (-53,-52,32). Activation was then extracted from these ROIs for the two tasks in the first study. The generalized least-squares model was implemented (R Statistical Package v2.15; R Development Core Team, 2008) to determine the best model fit for recognition rates with the gls function from the nlme package using restricted maximum likelihood estimation and compound symmetry for paired data (Pinheiro and Bates, 2009). The model examined the effects contour and task have on BOLD response (GLM beta values) with participant included as correlation factor: [gls(BOLD ~ Contour * Task, correlation = corCompSymm (form = ~1|Subject), data = D)]. Results are indicated using the interaction effect degrees of freedom, *t*-value, *p*-value, and Spearman’s rank correlation coefficient (rho).

## RESULTS

**FIGURE 2:**
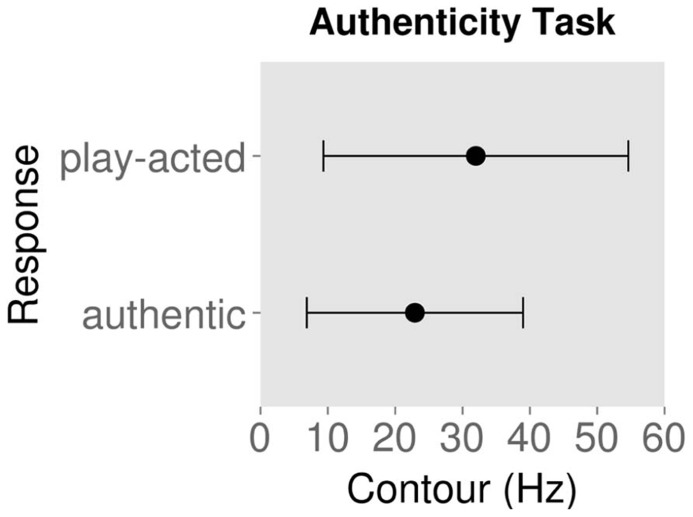
**Mean contour (measured as standard deviation of fundamental frequency) and standard deviation for behavioral responses to rating stimuli as authentic or play-acted**.

**FIGURE 3:**
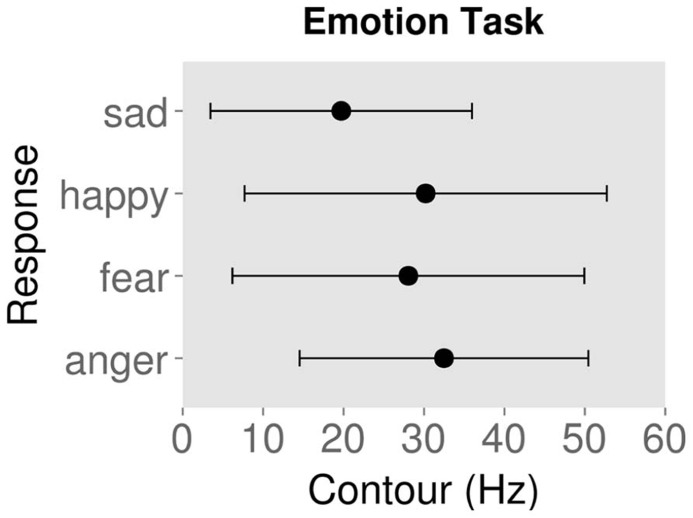
**Mean contour (measured as standard deviation of fundamental frequency) and standard deviation for behavioral responses to rating stimuli by emotion category**.

**Figures 2** and **3** show the mean ± standard deviation of contour values for each response type for the emotion and authenticity tasks respectively. Examining the behavioral effects of contour indicated that contour values for “authentic” responses were significantly lower than for “play-acted” responses [χ^2^(1) = 88.3, *p* < 0.001; **Figure 2**; **Table 2**]. In addition, contour values for “sad” responses were significantly different from “anger” [χ^2^(1) = 768.0, *p* < 0.001], “fear” [χ^2^(1) = 1037.9, *p* < 0.001], and “joy” [χ^2^(1) = 1089.6, *p* < 0.001], with stimuli labeled as “sad” being significantly lower in contour than the other emotions (**Figure 3**; **Table 2**).

**Table 2:** Mean values of high and low stimulus groups by F0 contour (SD).

		RT (s)	Emotion	Authenticity
Study 1	High contour	3.30 ± 0.59	0.46 ± 0.25	0.61 ± 0.29
	Low contour	3.12 ± 0.92	0.40 ± 0.31	0.70 ± 0.23
	Statistic	*t* = 0.91; *p* > 0.1	*t* = 0.93; *p* > 0.1	*t* = -1.18; *p* > 0.1
Study 2	High contour	3.09 ± 0.60	0.41 ± 0.24	
	Low contour	2.93 ± 0.82	0.47 ± 0.31	
	Statistic	*t* = 0.88; *p* > 0.1	*t* = -0.84; *p* > 0.1	

*Reaction time (in seconds) with SD and recognition rate (probability of correct recognition) with SD. Statistics represent *t*-tests of the differences in reaction times and recognition rates between the pre-selected contour groups.*

**FIGURE 4:**
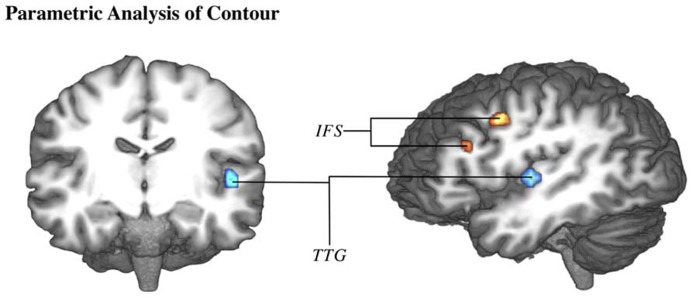
**Brain activation correlates of emotion experimental task of study 2 (Drolet et al., 2013)**. Group-averaged (*n* = 18) statistical maps of significantly activated areas for parametric effect of contour (higher contour in blue, lower contour in red). Posterior probability maps with a threshold of 99.5% and 100 voxel minimum size. Activation was mapped onto the best average fit subject 3D anatomical map. Left: anterior coronal section through middle temporal lobe. Right: left view sagittal section through temporal lobe. IFS, inferior frontal sulcus; TTG, transverse temporal gyrus.

The parametric analysis of brain activation with contour value was performed on the data for both studies. The parametric analysis of the first study was performed both across and split by task type, however, neither of these contrasts indicated any significant activation. Within the second study (which implemented only the emotion task), this analysis did produce significant activation in TTG (-47,-16,6) and inferior frontal sulcus (IFS: -41,-1,39; -38,23,24) (**Figure 4**).

**FIGURE 5:**
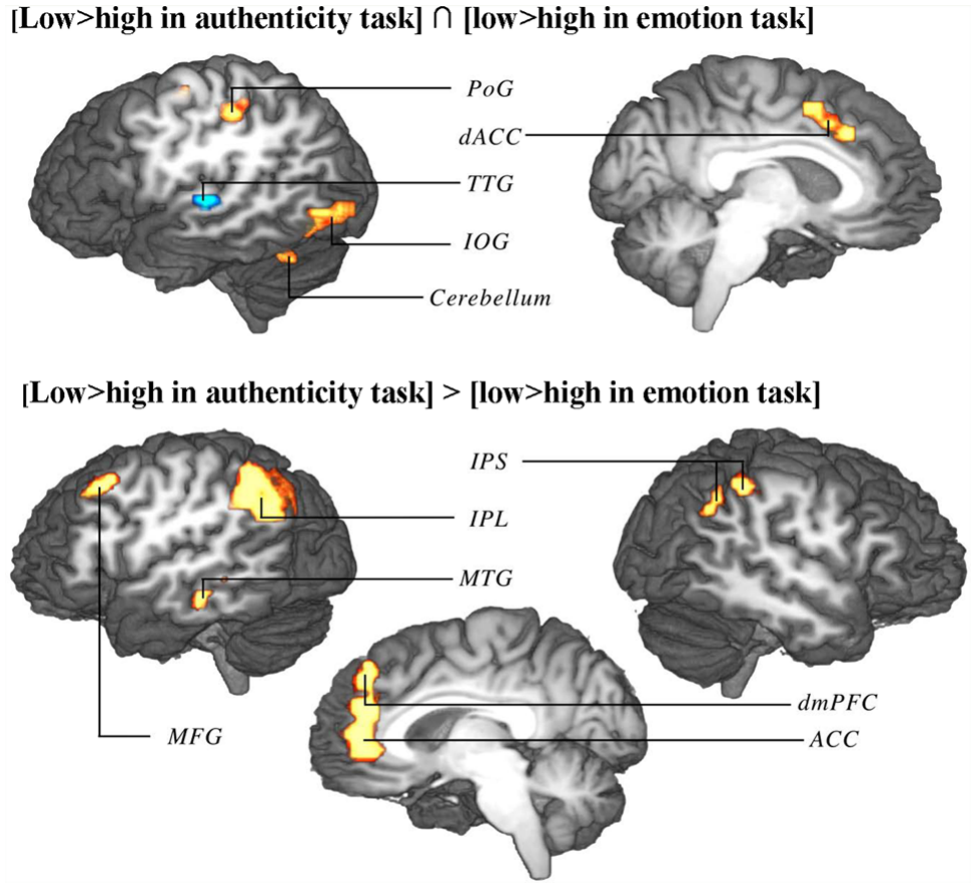
**Conjunction (top) and disjunction (bottom) of brain activation correlates of contour in authenticity task trials of study 1 and emotion task trials of study 2.** Top: regions marked red up-regulated by low contour in both tasks, regions marked blue up-regulated by high contour in both tasks. Bottom: interaction of contour extremes and task type (regions up-regulated by one end of the contour range in one task and by the other end of the contour range in the other task). Shown are group-averaged statistical maps (study 1: *n* = 24; study 2: *n* = 18). Posterior probability maps with a threshold of 99.5% and 100 voxel minimum size. Activation was mapped onto the best average fit subject 3D anatomical map. Top: left view sagittal section. Bottom: left view sagittal section through midline. PoG, postcentral gyrus; dACC, dorsal anterior cingulate cortex; TTG, transverse temporal gyrus; IOG, inferior occipital gyrus; IPS, inferior parietal sulcus; IPL, inferior parietal lobule; MTG, middle temporal gyrus; MFG, middle frontal gyrus.

Due to reduced contour effects for the emotion task in study 1, whole-brain contour-induced activations during authenticity and emotion trials could only be compared between studies 1 and 2 (not within study 1). Both a conjunction and an exclusive disjunction were performed on these two contrasts. The conjunction (regions similarly modulated in both tasks) revealed activation in the precentral (PrG) and postcentral gyri (PoG) on the left, the dorsal anterior cingulate cortex bilaterally (dACC) extending into the left pre-supplementary motor area (pre-SMA), the left TTG, the left inferior occipital gyrus (IOG), and the lateral hemispheres of the cerebellum. The disjunction (regions differently modulated between tasks) revealed activation in the dorsomedial prefrontal cortex bilaterally (dmPFC) extending into anterior cingulate cortex (ACC) bilaterally, the left middle frontal cortex (MFG), the left inferior parietal lobule (IPL), the right inferior parietal sulcus (IPS), and left MTG (**Table 3**; **Figure 5**).

**Table 3:** Coordinates of conjunction and disjunction of brain activation correlates of contour in authenticity task trials of study 1 and emotion task trials of study 2.

			Talairach coordinates		
Area	BA	Hemisphere	*x*	*y*	*z*
**Conjunction**
SMA	6	L	-5	6	48
dACC	32	L	-8	21	36
	32	R	1	21	36
PrG	6	L	-29	-9	54
PoG	3	L	-41	-28	51
TTG	42	L	-53	-15	6
IOG	19	L	-47	-78	0
	37	L	-44	-63	-3
Cerebellum	Crus I	L	-41	-54	-21
Cerebellum	V	R	13	-51	-12
**Disjunction**
mPFC	10	L	-11	47	12
	9	R	1	41	24
ACC	32	L	-5	38	21
	32	R	4	41	3
MFG	8	L	-38	24	45
IPL	40	L	-56	-48	42
IPS	40	R	49	-36	42
MTG	21	L	-65	-24	-9

*Conjunction (regions similarly modulated by contour in either task) and disjunction (interaction: regions differentially modulated in either task) of brain activation correlates of contour in authenticity task trials of study 1 and emotion task trials of study 2 (study 1: *n* = 24; study 2: *n* = 18). Anatomical specification, Brodmann area (BA) Talairach coordinates of local maxima (>99.5% Bayesian probability). aMCC, anterior middle cingulate; MFG, middle frontal gyrus; SPL, superior parietal lobule; IPL, inferior parietal lobule; FG, fusiform gyrus; IOG, inferior occipital gyrus; sgACC, subgenual anterior cingulate; mPFC, medial prefrontal; SFG, superior frontal gyrus; STG, superior temporal gyrus; TTG, transverse temporal gyrus; pSTS, posterior superior temporal sulcus; TPJ, temporoparietal junction; pSTS, posterior superior temporal sulcus; TPJ, temporoparietal.*

**FIGURE 6:**
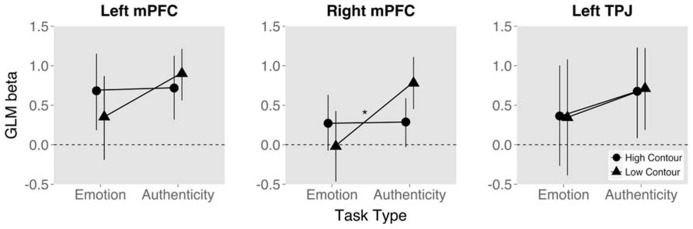
**Mean and 95% confidence interval of BOLD signal measure (GLM beta value) by task type and trial stimulus fundamental frequency contour value.** Measures from three regions of interest selected from study 2 and extracted within study 1. (*p < 0.05).

Finally, the ROI analysis performed on the data from the first study, split by task, using coordinates extracted from the second study, showed a clear interaction effect of task and contour. While high contour up-regulated TTG and STG activation independent of task, bilateral activation in mPFC appeared to increase for low contour during the authenticity task and high contour during the emotion task. Of these tendencies, the activation in right mPFC reached significance, such that the interaction of contour and task was significant [*t*(84) = 3.26, *p* < 0.01, ρ = 0.604; **Figure 6**].

## DISCUSSION

Independent of the task, high contour stimuli (which are more likely to be play-acted than authentic) induced BOLD modulation in the left primary auditory cortex or TTG. Within the cortex, initial modulation of activation by auditory stimulation occurs in TTG (Celesia, 1976; Arnott et al., 2004). Therefore, contour has a strong effect on activation even at a very early stage in perception and can be considered the source of the authenticity effects seen in the primary auditory cortex (Drolet et al., 2013). Notably, stimuli with high variability of F0 frequencies (i.e., high contour) stimulated a larger portion of the TTG than stimuli with low variability. This is due to the fact that frequencies are mapped tonotopically in TTG (Romani et al., 1982; Pantev et al., 1995), such that segments within the TTG are sensitive to different sound frequencies. The activation of a greater area within the cortex leads to a greater BOLD response. Interestingly, participant behavior was affected by this sensitivity to F0 contour, as seen in the correlation between responses to explicit categorization tasks and contour (**Figure 1**). While the range of contour values in these correlations was quite large and their corresponding effects are subtle, “authentic” and “sad” responses were nevertheless associated with lower contour values than “play-acted” or other emotions, respectively.

While activation in TTG was left-lateralized, any conclusions on the role of such lateralization would be speculative. There is much research indicating a right-lateralization for emotion perception in speech (Johnsrude et al., 2000; Zatorre and Belin, 2001), but there is also evidence to the contrary (Ethofer et al., 2009; Grimshaw et al., 2009). Clearly, hemispheric differences are an active area of research and several non-hypothesis driven reverse inferences could be valid in this context. More important to this study, fMRI BOLD analyses can exaggerate the influence of laterality differences. While Bayes statistics are not influenced by differences in *p*-values, comparing activation patterns between brain regions would represent a non-statistical comparison of probabilities. Therefore, these effects will not be discussed in detail here.

Low variability of F0 frequencies induced a very different pattern of activation. Task-independent activation (conjunction) by low contour stimuli (which are more likely to be authentic than play-acted) included dorsal ACC, extending into pre-SMA. Activation in ACC was seen in previous work (Drolet et al., 2012, 2013). The novel finding presented here is that this ACC activation was associated specifically with low contour stimuli. ACC activation has been attributed to overall task difficulty (Ridderinkhof et al., 2004). As suggested by Volz et al. (2005), and further corroborated by Pochon et al. (2008), activation in medial SMA and dorsal ACC is modulated relative to difficulty distinguishing stimuli themselves while the task is simple (response conflict), as opposed to complex tasks and difficult rules related to task completion (decision conflict). The high and low contour stimuli in the current study were more likely to be play-acted and authentic respectively, but the emotion categories were not equally distinct at either extremes of the contour range: the difference between the lowest contour values of authentic and play-acted stimuli was only 1.31 Hz, while the difference between the highest values was 22.96 Hz (data from Jürgens et al., 2011), such that the authentic versus play-acted distinction was much clearer between high contour stimuli. The increased ambiguity in distinguishing authentic and play-acted between low contour stimuli likely induced the increased BOLD response in ACC.

The disjunction, on the other hand, showed that medial BA 9 (bilaterally extending into anterior ACC) was differentially activated between the tasks. Activation increased for low contour values during the authenticity task, while they did so for high contour values during the emotion task. Activation in mPFC and TPJ was previously seen in study 1 (Drolet et al., 2012) as part of the ToM network (Frith and Frith, 2003), suggesting that contour is the source of the previously seen authenticity effects in mPFC (Drolet et al., 2012). In that study we hypothesized that mPFC may generally be activated for rating authenticity as part of the ToM network. The current results more specifically indicate that during explicit rating of authenticity the ambiguous low contour stimuli induced activation in mPFC. This was additionally corroborated via the direct comparison of values from emotion and authenticity tasks using ROIs from study 2 within study 1 (focusing specifically on TPJ and mBA9). Within these previous coordinates, right mPFC activation was significantly different for low contour stimuli. While mPFC was recruited in the case of a ToM-relevant task such as the rating of stimulus authenticity, this activation was up-regulated specifically for ambiguous stimuli that were more difficult to perceive and may have therefore required more resources to distinguish. This differentiation also reemphasizes the importance of contour in perception of authenticity since stimuli with ambiguous contour did not induce activation during the emotion task.

The remaining sites of the network uncovered by the disjunction were part of the top-down attention control network. For example, Hopfinger et al. (2000) found that the superior frontal gyrus (SFG), parietal, and occipital gyri increased activation during top-down shifting of attention, potentially due to increased working memory load. The novel result shown in the disjunction was that, while this task-related attention occurred toward low contour stimuli during authenticity rating, the emotion task actually recruited more resources for high contour stimuli. While the latter may have been due to the bottom-up stimulation of TTG by high contour stimuli, the perception of play-acting may also have been perceived as a form of pretense, inducing increased activation to ensure that the correct emotion was perceived relative to the context at hand (German et al., 2004; Shany-Ur et al., 2012). The involvement of the perception of pretense in the use of acted behavior in similar research should be examined further considering the importance of such stimuli in emotion research. In fact, Altmann et al. (2012) found that reading invented versus real stories activated regions including the dACC and TPJ, indicating that this network is particularly important in differentiating between sources of the information.

The fact that not only phylogenetically older structures, such as the primary cortices, but also higher association cortices (e.g., TPJ) and frontal sites (e.g., PFC) are engaged during decoding of expressed emotions, points to a complex interplay of automatic and cognitively reflected components. The findings presented here concur with theoretical approaches that include the cognitive evaluation of the surrounding event in emotion perception (Scherer, 1984; Smith and Ellsworth, 1985; Frijda, 1986; Lazarus, 1991).

The current results and previous findings (Drolet et al., 2012, 2013) also point to an intriguing functional difference between TPJ and mPFC. TPJ was recruited for explicit rating of authenticity or when a stimulus was labeled as either authentic or play-acted, but this activation did not interact with bottom-up stimulus perception (Drolet et al., 2012, 2013). mPFC, however, was up-regulated by low contour (authentic) stimuli when explicitly rating stimulus authenticity. While confirming the previous hypothesis that mPFC activation by stimulus categories is dependent on task (Drolet et al., 2013), it is now clear that this interaction is specific to the feature of greatest importance to that task: As stimuli became more ambiguous due to less distinct F0 contour, the mPFC required more resources to rate their authenticity. While the TPJ was activated by both task requirements and stimulus features, no interaction occurred, leaving bottom-up/top-down integration to the mPFC.

Finally, considering the question of how emotions are to be defined, the results presented here support the view that the perception of emotions is a multi-component phenomenon. While it is known that acoustic features indicate emotional content, we have provided evidence that prosodically encoded authenticity can interact with emotional information. The integration of these features represents a novel complex interaction that can be observed both behaviorally (Jürgens et al., 2013) and in BOLD responses related to the implicit and explicit perception of these acoustic features. As the effect can occur independent of the awareness of authenticity (Drolet et al., 2012), and therefore cannot be easily dissociated from emotion perception itself, we suggest that this process is one of the many integral components of emotion perception.

## Conflict of Interest Statement

The authors declare that the research was conducted in the absence of any commercial or financial relationships that could be construed as a potential conflict of interest.
